# Design and Performance Analysis of a Compact Planar MIMO Antenna for IoT Applications

**DOI:** 10.3390/s21237909

**Published:** 2021-11-27

**Authors:** Saminathan Thiruvenkadam, Eswaran Parthasarathy, Sandeep Kumar Palaniswamy, Sachin Kumar, Lulu Wang

**Affiliations:** 1 Department of Electronics and Communication Engineering, SRM Institute of Science and Technology, Kattankulathur, Chennai 603203, India; saminatt@srmist.edu.in (S.T.); eswaranp@srmist.edu.in (E.P.); gupta.sachin0708@gmail.com (S.K.); 2 Biomedical Device Innovation Center, Shenzhen Technology University, Shenzhen 518118, China

**Keywords:** antenna, IoT, MIMO, monopole, smart home

## Abstract

This article presents a quad-band multiple-input-multiple-output (MIMO) antenna for the Internet of Things (IoT) applications. The proposed antenna consists of four quarter-wavelength asymmetrical meandered radiators, microstrip feed lines, and modified ground planes. The antenna elements are arranged in a chiral pattern to improve isolation between them, with two radiators and two ground planes placed on the front side of the substrate and the other two on the back side. The MIMO antenna has an operating bandwidth (S_11_ ≤ −10 dB) of 1.76–1.84 GHz, 2.37–2.56 GHz, 3.23–3.68 GHz, and 5.34–5.84 GHz, covering GSM, WLAN, WiMAX, and 5G frequency bands. The isolation between the radiating elements is greater than 18 dB in the operating bands. The peak gain of the antenna is 3.6 dBi, and the envelope correlation coefficient (*ECC*) is less than 0.04. Furthermore, the proposed antenna is validated for IoT-based smart home (SH) applications. The prototype MIMO antenna is integrated with a commercially available ZigBee device, and the measured values are found to be consistent with the expected results. The proposed MIMO antenna could be a good candidate for IoT systems/modules due to its low profile, compact size, lightweight, and easy integration with wireless communication devices.

## 1. Introduction

The Internet of Things (IoT) is a fast-growing technology that connects physical objects, household appliances, wearable devices, sensors, servers, and other wired or wireless networks [1]. These devices can process data and send it to each other without manual intervention. IoT infrastructure is used in a variety of industries, such as healthcare, transportation, agriculture, and smart cities [2]. Future communications will be coordinated by systems integrated with IoT technology, and such communication devices will require multi-band antennas to support various frequencies, standards, and applications.

Various types of multi-band antennas, such as dipole, patch, monopole, and dielectric resonator, have been proposed in the literature [3,4,5,6,7,8,9,10,11,12]. For WLAN/WiMAX applications, E-shaped [8], slotted triangular-shaped [9], asymmetric T-shaped [10], half mode substrate integrated cavity [11], and coplanar waveguide (CPW)-fed rectangular-shaped [12] dual-band antenna structures were presented. Several triple-band monopole antenna structures were also reported in the literature [13,14,15,16,17,18,19,20]. A Y-shaped radiator with a dual ring resonator [13], a Y-shaped radiator with an L-shaped slit etched from the ground plane [14], a rectangular patch integrated with a step-shaped microstrip feed line [15], L-shaped and T-shaped radiators [16], a T-shaped resonator with an open slot etched from the ground plane [17], rectangular patch with two symmetrical E-shaped slots etched from the ground plane [18], multibranch strips [19], and overlapping strips [20] were proposed for LTE, WiMAX, and WLAN applications. In the literature, a few quad-band monopole antenna configurations were also investigated [21,22]. Inverted L-shaped monopole with microstrip line feeding [21] and meandered line-based monopole [22] antennas were presented for LTE, WLAN, WiMAX, and INSAT-C applications.

In recent years, a few multiple-input-multiple-output (MIMO) antenna structures for IoT applications have also been reported [23,24,25,26,27,28,29,30,31,32,33,34,35,36]. A triple-band MIMO antenna with a complementary split-ring resonator was presented in [23]. A planar multi-band MIMO antenna in [24], a two-port dielectric resonator antenna structure in [25], a meander line radiator loaded with a split ring resonator in [26], an ellipse-shaped multi-band MIMO antenna in [27], an eight-port meandered line structure in [28], a four-port MIMO antenna for 5G applications in [29], a four-port L-shaped planar inverted-F antenna in [30], a two-port dielectric resonator antenna for LTE applications in [31], a CPW-fed sickle-shaped resonator for IoT application in [32], a meander line monopole antenna for multiband applications in [33], a monopole antenna with vias for IoT applications in [34], a CPW-fed rectangular patch radiator in [35], and a combination of simple monopole radiators for IoT/WLAN/sub-6 GHz/X-band applications in [36]. However, the majority of the above-reported MIMO antenna designs were large in size and difficult to integrate on the printed circuit board of the IoT module.

In this article, a four-port quad-band double-sided (DS) MIMO antenna is presented for IoT applications. The antenna radiators in the DS MIMO antenna are located on the front and back sides of the dielectric substrate in order to reduce mutual coupling. Multiple strips of different wavelengths are integrated with the radiator to achieve four different (GSM, Wi-Fi/WLAN, WiMAX, and 5G) frequency bands. A conical ground plane is used to obtain impedance matching at the desired frequencies. The size of the proposed DS MIMO antenna is 60 mm × 60 mm. Furthermore, real-time verification of the DS MIMO antenna for an IoT-based smart home system is performed.

## 2. Antenna Design

**Figure 1:**
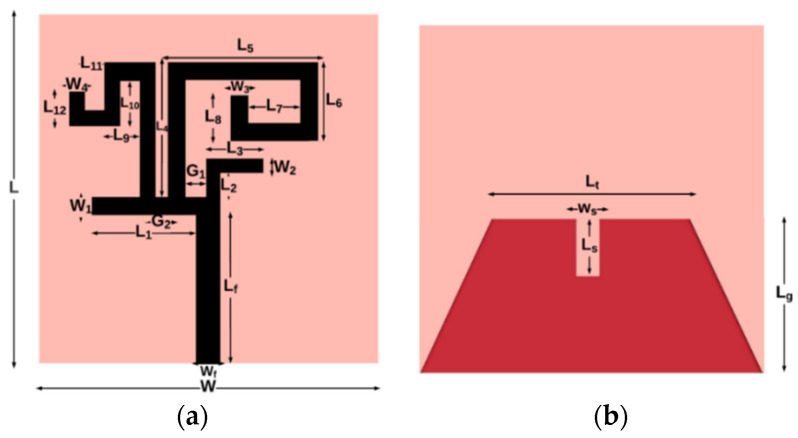
Proposed antenna element (**a**) front side (radiator), (**b**) back side (ground plane).

The top view and bottom view of the proposed antenna element are shown in Figure 1a,b, respectively. The antenna element consists of a radiator, composed of four metal strips of varying lengths, a microstrip line feed, and a tapered ground plane. The proposed monopole antenna is fabricated on the FR-4 substrate of relative permittivity of 4.4 and thickness of 1.6 mm. The antenna element and MIMO antenna are simulated using the CST Microwave Studio^®^ software, and the size of the antenna element is 30 mm × 20 mm. The dimensions of the quad-band antenna are depicted in Table 1.

### 2.1. Evolution of the Antenna Element

**Figure 2:**
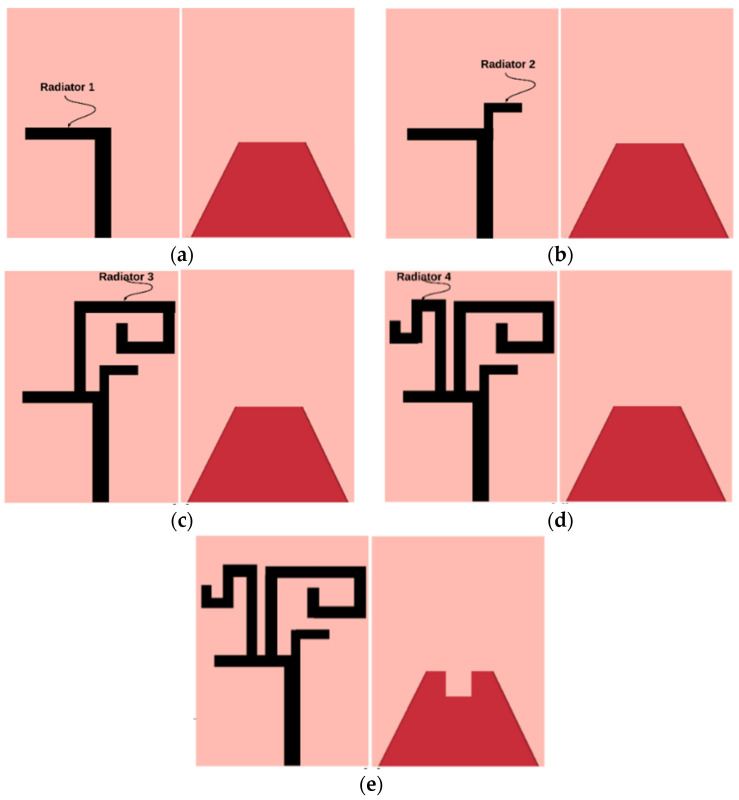
Development of the quad-band antenna element (**a**) antenna-1, (**b**) antenna-2, (**c**) antenna-3, (**d**) antenna-4, (**e**) proposed antenna.

**Figure 3:**
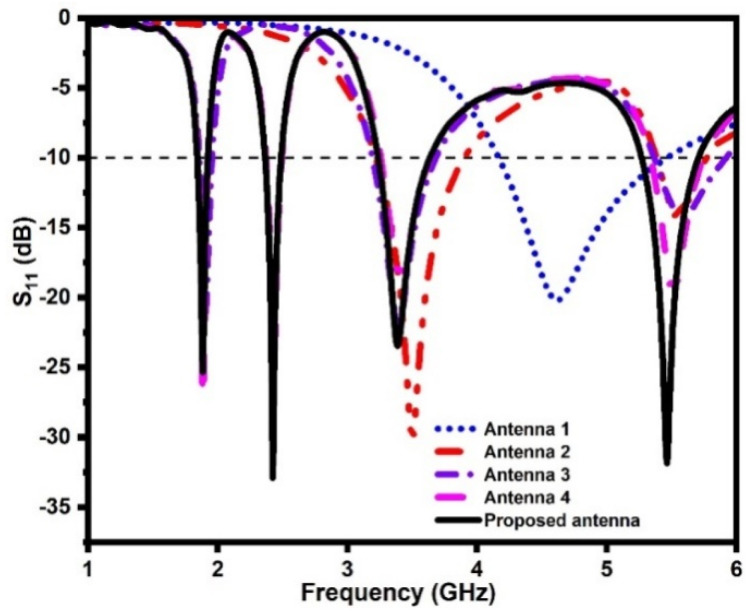
Reflection coefficients of the quad-band antenna design stages.

The development of the quad-band antenna element is illustrated in Figure 2. The length of the resonator can be calculated using Equation (1):

(1) $$\;f_{ri}=\frac{C}{4L_{sj}\sqrt{\varepsilon_{eff}}};\;i,\;j=1,\;2,\;3,\;4$$

(2) $$\varepsilon_{eff}=\frac{\varepsilon_{r+1}}{2}$$

where *C* is the velocity of light in vacuum, *f_ri_* is the resonating frequency, and *ɛ_eff_* is the effective dielectric constant of the substrate. The antenna-1 is composed of a monopole radiator (radiator-1) integrated with a 50 Ω microstrip feed line and a tapered ground plane, as depicted in Figure 2a. The reflection coefficients of the quad-band antenna design steps are depicted in Figure 3. It is noticed that the antenna-1 resonates at 4.9 GHz.

In step-2, a rotated L-shaped stub (radiator-2) is integrated with the antenna-1, as shown in Figure 2b. The antenna-2 offers dual-band resonance at frequencies of 3.5 GHz and 5.5 GHz, as illustrated in Figure 3. Next, a folded meandered line stub (radiator-3) is integrated with the antenna-2, as illustrated in Figure 2c.

The antenna-3 resonates at 1.8 GHz, 3.5 GHz, and 5.5 GHz. In the next step, as illustrated in Figure 2d, one more meandered line stub (radiator-4) is integrated with the antenna-3. This stub adds an extra resonance at 2.4 GHz. The antenna-4 is a quad-band antenna as it resonates at 1.8 GHz, 2.4 GHz, 3.5 GHz, and 5.5 GHz.

In the design process, the distance between radiators-1, -2, -3, and -4 is optimized to achieve the minimum coupling between them. Furthermore, a U-shaped slot is etched from the tapered ground plane to improve impedance matching at the resonating frequency bands, as illustrated in Figure 2e.

**Figure 4:**
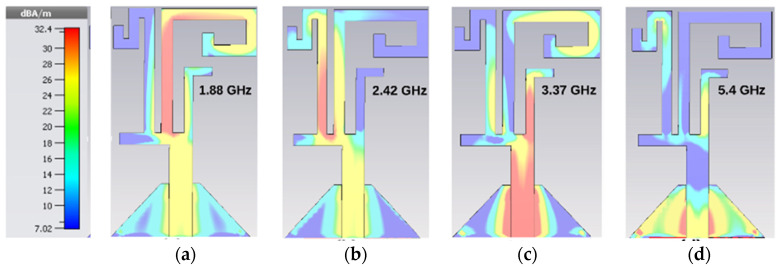
Surface current distribution at (**a**) 1.88 GHz, (**b**) 2.42 GHz, (**c**) 3.37 GHz, (**d**) 5.4 GHz.

Figure 4 shows the current distribution at four resonances, which validates the antenna’s multiband behavior. The current distribution at the first resonance is illustrated in Figure 4a. It is obvious that the radiator-3 has the highest current density at 1.88 GHz. The current distribution at the second resonance is illustrated in Figure 4b. It can be noticed that the radiator-4 has the highest current density at 2.42 GHz. The current distribution at the third resonance is depicted in Figure 4c. It is apparent that the radiator-2 has the highest current density at 3.37 GHz. The current distribution for the fourth resonance is depicted in Figure 4d. It is observed that the radiator-1 has the lowest current flow at 5.4 GHz, and radiators-2 and -3 have the highest current distribution due to their connection to radiator-1. The current distribution confirms that the antenna operates in multiple bands and independently.

### 2.2. MIMO Implementation

**Figure 5:**
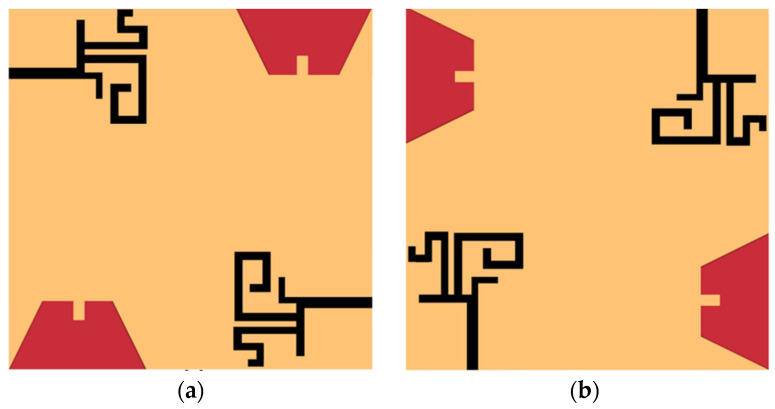
Proposed DS MIMO antenna (**a**) front side, (**b**) back side.

**Figure 6:**
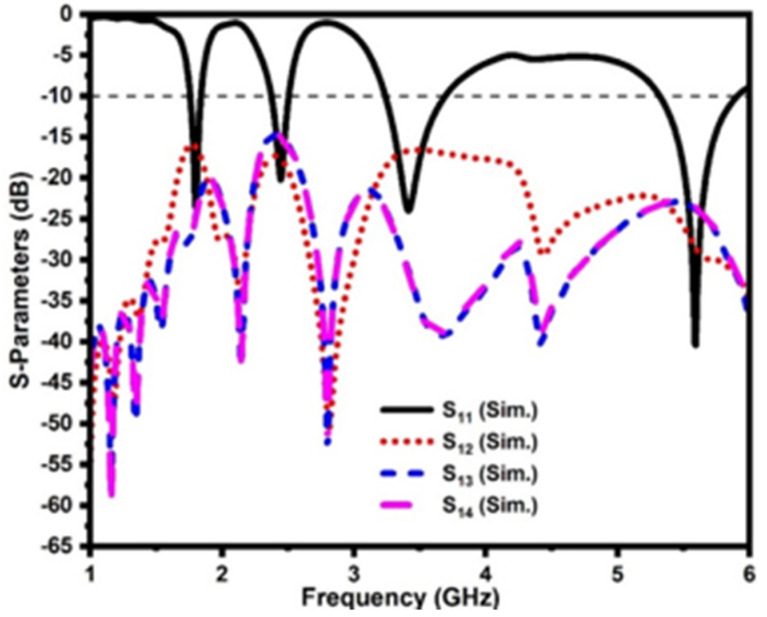
Simulated S−parameters of the proposed DS MIMO antenna.

The front view and back view of the proposed DS MIMO antenna are shown in Figure 5. The proposed MIMO antenna employs the double-sided radiator placement method to improve antenna element isolation. Two radiators and two ground planes are located on the front side of the substrate, as shown in Figure 5a, and the other two radiators/ground planes on the back side, as shown in Figure 5b. The four identical antenna elements are also arranged orthogonally to one another. The size of the DS MIMO antenna is 60 mm × 60 mm. The simulated reflection coefficients of the DS MIMO antenna are illustrated in Figure 6. The proposed MIMO antenna resonates at 1.88 GHz, 2.42 GHz, 3.37 GHz, and 5.4 GHz, and the isolation between antenna elements is more than 15 dB. It is evident that the double-sided placement of the antenna elements improves isolation significantly.

**Figure 7:**
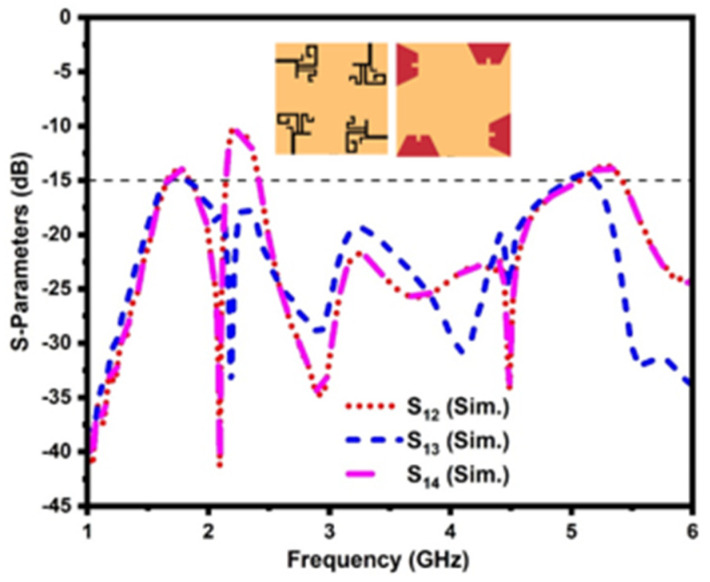
Mutual coupling of the conventional MIMO antenna.

Whereas in conventional MIMO antenna design, the four radiating elements are placed on the same side of the dielectric substrate, and the isolation between them is not impressive, even though the radiators are orthogonal to each other, as illustrated in Figure 7. The conventional MIMO antenna has a mutual coupling of less than −15 dB and −10 dB in the operating bands.

## 3. Results and Discussion

**Figure 8:**
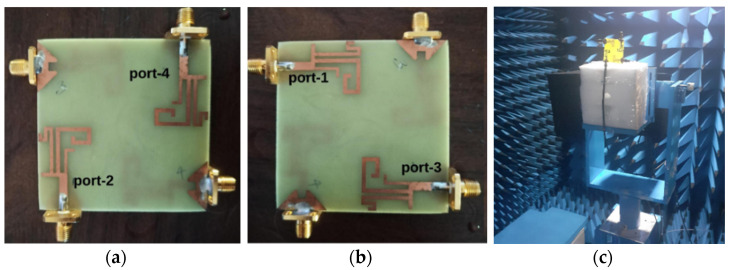
Prototype of the DS MIMO antenna (**a**) front view, (**b**) back view, (**c**) measurements in an anechoic chamber.

**Figure 9:**
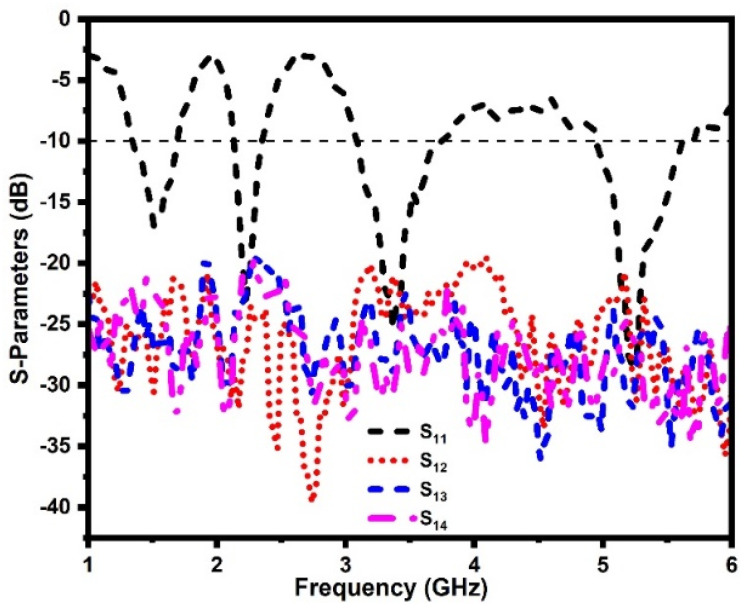
Measured S−parameters of the proposed antenna.

Figure 8a–c shows photographs of the DS MIMO antenna prototype and measurements in an anechoic chamber. The S-parameters are measured using the N9926A vector network analyzer. Figure 9 shows the measured reflection coefficients of the proposed DS MIMO antenna. It can be observed that the antenna operates at 1.7 GHz, 2.3 GHz, 3.4 GHz, and 5.4 GHz. It is also noticed that the measured isolation is more than 20 dB at the operating bands.

**Figure 10:**
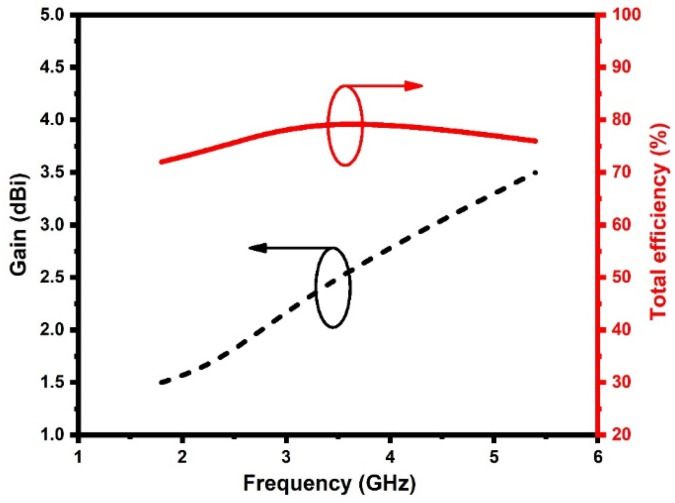
Measured gain and efficiency of the DS MIMO DS MIMO antenna.

Figure 10 represents the measured gain and efficiency of the DS MIMO antenna. The antenna gain varies from 1.5 to 3.6 dBi, and the efficiency varies from 72 to 81%. The peak gain and efficiency achieved at 1.8 GHz, 2.4 GHz, 3.4 GHz, and 5.4 GHz are 1.5 dBi (72%), 2.5 dBi (75%), 2.8 dBi (81%), and 3.6 dBi (76%), respectively.

**Figure 11:**
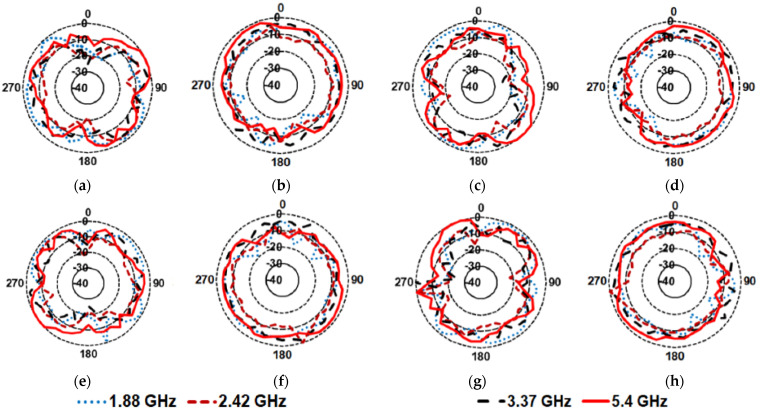
Measured radiation patterns of the DS MIMO antenna (**a**) antenna−1, E−plane, (**b**) antenna−1, H−plane, (**c**) antenna−2, E−plane, (**d**) antenna−2, H−plane, (**e**) antenna−3, E−plane, (**f**) antenna−3, H−plane, (**g**) antenna−4, E−plane, (**h**) antenna−4, H−plane.

Figure 11 presents the measured radiation patterns of the DS MIMO antenna at 1.88 GHz, 2.42 GHz, 3.37 GHz, and 5.4 GHz. It is observed that the radiation patterns are nearly omnidirectional at all operating frequencies, indicating that the proposed MIMO antenna is well suited for IoT applications.

The MIMO performance metrics of the DS MIMO antenna are evaluated in terms of envelope correlation coefficient (*ECC*), diversity gain (*DG*), total active reflection coefficient (TARC), channel capacity loss (CCL), mean effective gain (MEG), cumulative distribution function (CDF).

### 3.1. ECC

*ECC* is one of the key parameters of a MIMO antenna system. It investigates the interference between the antenna elements when all of them are excited simultaneously [37]. The *ECC* can be evaluated using the following formula [7].

(3) $$ECC=\frac{{\left|\iint \left[\vec{F_{a}}\left(\theta ,\varphi \right).\vec{F_{b}}\left(\theta ,\varphi \right)\right]d\Omega \right|}^{2}}{\iint {\left|\vec{F_{a}}\left(\theta ,\varphi \right)\right|}^{2}d\Omega \;\iint {\left|\vec{F_{b}}\left(\theta ,\varphi \right)\right|}^{2}d\Omega }$$

where *F_i_*(θ,φ) is the radiated field of the *i*th antenna, and *θ*, *ϕ*, and Ω are the elevation, azimuthal, and solid angles, respectively. Table 2 shows that the measured *ECC* of the proposed DS MIMO antenna is <0.04 in the operating bands. It confirms that the MIMO antenna elements have a low correlation.

### 3.2. DG

The *DG* can be computed by using Equation (4).

(4) $$DG=10\sqrt{1-{\left|\rho_{eij}\right|}^{2}}$$

where $\rho_{eij}$ is the value of *ECC* obtained using the far-field radiation pattern. The measured *DG* values of the proposed DS MIMO antenna are shown in Table 2. In the operating bands, the *DG* is greater than 9.8 dB.

### 3.3. MEG

MEG is one of the most significant metrics for analyzing the performance of MIMO antennas. Under multipath fading conditions, the MEG is defined as the ratio of the received mean power of the diversity antenna to the received mean power of the isotropic antenna [38]. The MEG for the proposed diversity antenna is calculated using Equation (5). The MEG values for the proposed DS MIMO antenna are around one, as shown in Table 2.

(5) $$MEG_{i}=0.5\;\left(1-\overset{N}{\underset{j=1}{\sum }}{\left|S_{ij}\right|}^{2}\right)$$

where *N* is number of antennas.

### 3.4. TARC

The adjacent radiating antennas affect the overall efficiency and operating bandwidth of the MIMO system [39]. In order to take this impact into account, a new metric TARC was introduced. The TARC can be calculated using Equation (6).

(6) $$TARC=\sqrt{\frac{{\left|S_{ii}+S_{ij}e^{j\theta }\right|}^{2}+{\left|S_{ji}+S_{jj}e^{j\theta }\right|}^{2}}{2}}$$

where *θ* is the phase angle, *S_ii_* and *S_jj_* are the reflection coefficients, and *S_ij_* and *S_ji_* are the transmission coefficients. Table 3 presents the measured TARC of the proposed DS MIMO antenna. In the operating bands, the TARC is less than −20 dB.

### 3.5. CCL

CCL describes the channel capacity loss caused by the correlation of radiators [40]. The CCL of the MIMO antenna must be <0.5 bits/Hz/s. The CCL can be evaluated using Equations (7) and (8).

(7) $$CCL=-\log_{2}\left|\psi^{R}\right|$$

where $\psi^{R}$ is the correlation matrix of the receiving antenna and is expressed as

(8) $$\;\psi^{R}=\left[\psi_{ii}\;\psi_{ij}\;\psi_{ji}\;\psi_{jj}\;\right]$$

where

(9) $$\psi_{ii}=1-\left({\left|S_{ii}\right|}^{2}+{\left|S_{ij}\right|}^{2}\right)$$

where,

(10) $$\;\psi_{jj}=\left(S_{ii}{}^{*}S_{ij}+S_{ji}{}^{*}S_{jj}\right)\;for\;i,\;j=1\;or\;2$$

**Figure 12:**
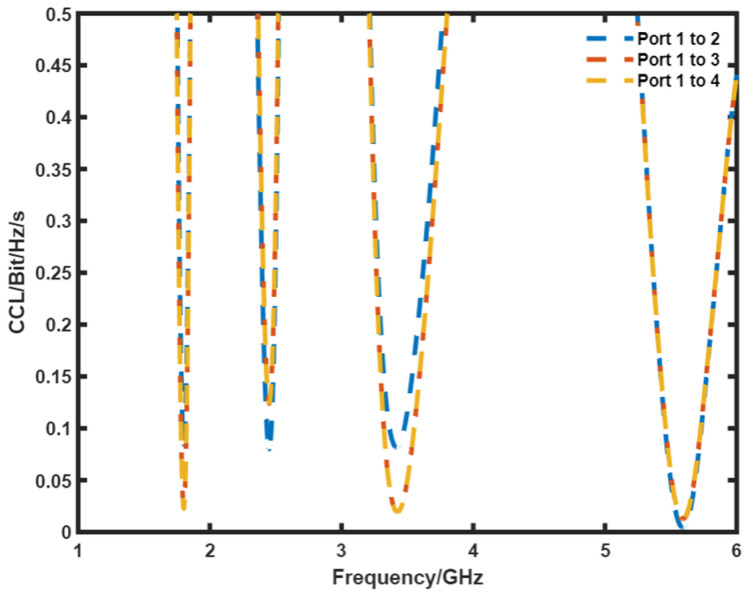
Measured CCL of the DS MIMO antenna.

Figure 12 presents the measured CCL of the proposed DS MIMO antenna. In the operating bands, the proposed antenna has a CCL of <0.28 bits/Hz/s.

### 3.6. CDF

**Figure 13:**
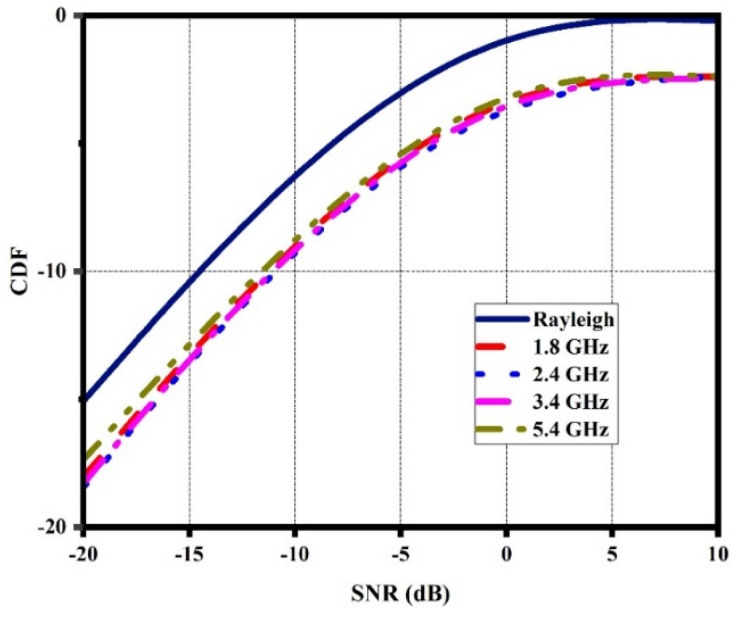
CDF of the DS MIMO antenna.

CDF is an important metric for evaluating the diversity performance of a quad-port MIMO antenna in the Rayleigh fading scenario. [41]. The CDF at various frequencies can be calculated using Equations (11) and (12). The CDF values of the DS MIMO antenna are shown in Figure 13.

(11) $$P_{MRC}\left(\gamma \leq X\right)=1-\overset{N}{\underset{i=1}{\sum }}\left(\frac{\lambda_{i}^{N-1}e^{\frac{-X}{\lambda_{i}}}}{\pi_{i\neq j}^{N}\lambda_{i}-\lambda_{j}}\right)\;$$

where *N* is the number of antenna elements and *λ* is the Eigen value obtained through the signal covariance matrix Ʌ_MRC_ obtained through $\rho_{e}$ and the MEG as given by Equation (12).

(12) $${Ʌ}_{MRC}=\rho_{e}\sqrt{MEG_{i\;}MEG_{j}}\;$$

### 3.7. Housing Effect

**Figure 14:**
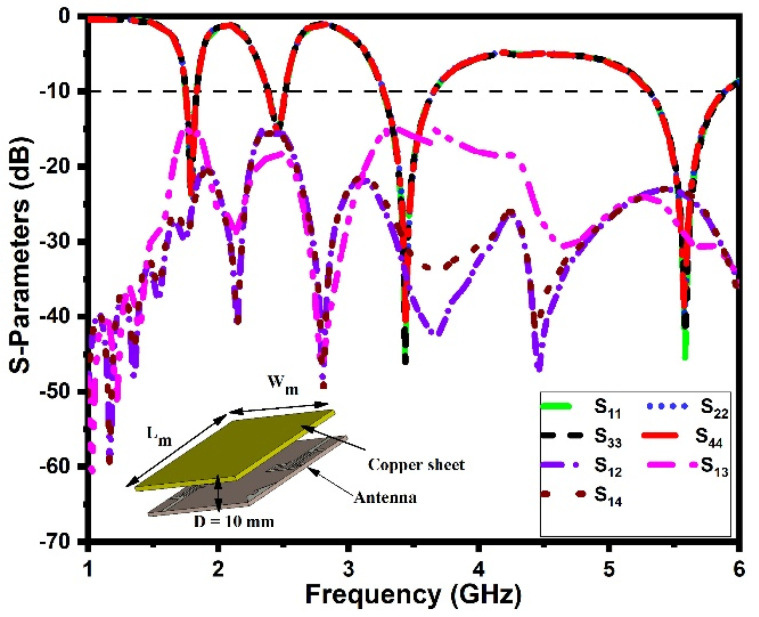
S−parameters under the copper sheet.

**Figure 15:**
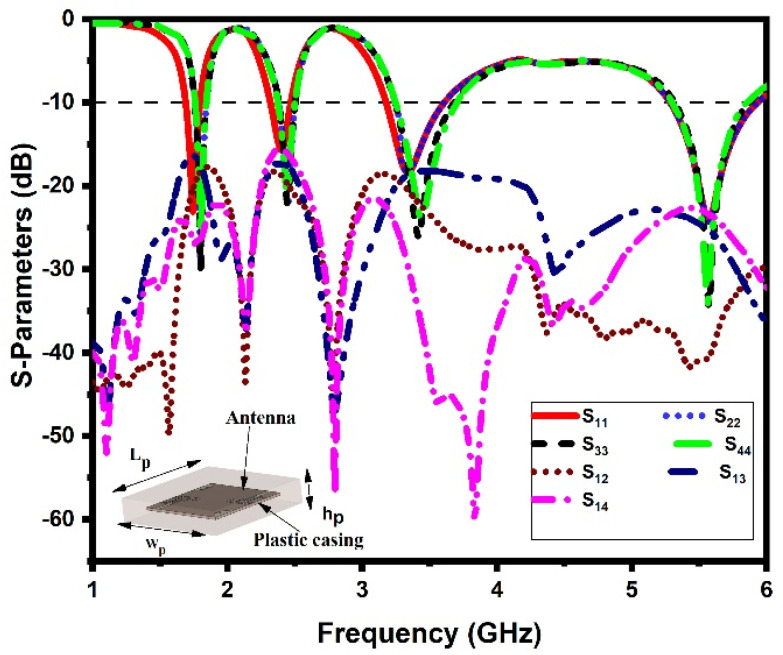
S−parameters inside the plastic casing.

The performance of the antenna should remain unchanged after mounting it on the host device. In this section, the antenna-in-packaging performance of the proposed DS MIMO antenna is investigated under two different scenarios. In the first scenario, the antenna is placed under a copper sheet (with dimensions of *L_m_* × *W_m_* = 60 mm × 60 mm) at a distance (*D*) of 10 mm, and in the second scenario, the antenna is placed inside a plastic casing (with dimensions of *L_p_* × *W_p_* × *h_p_* = 70 mm × 70 mm × 10 mm) [35,42]. The corresponding S-parameters (reflection coefficients and mutual coupling) of the proposed antenna in two different scenarios are shown in Figure 14 and Figure 15. It is noted that the proposed antenna exhibits quad-band performance even when placed under a copper sheet and plastic casing in the near-field region. Hence, the copper sheet and plastic casing have a minimal effect on antenna performance, confirming its stable working in IoT devices.

## 4. Real-Time Verification of the DS MIMO Antenna

**Figure 16:**
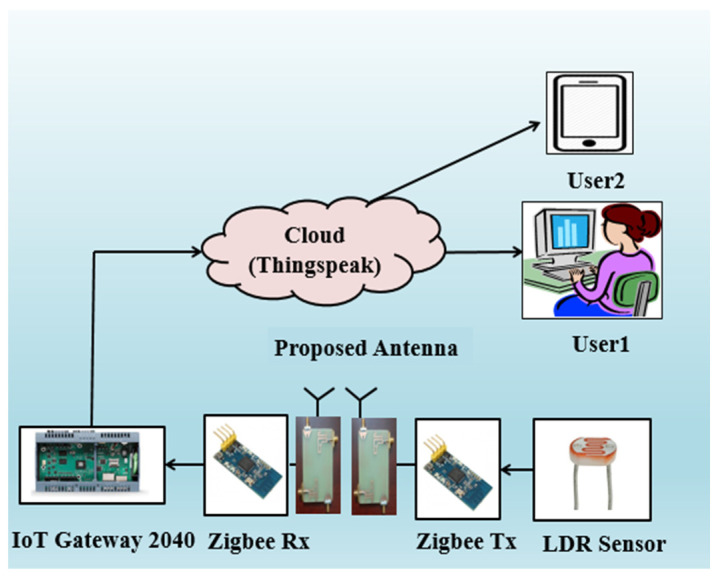
System architecture of the proposed smart home application.

**Figure 17:**
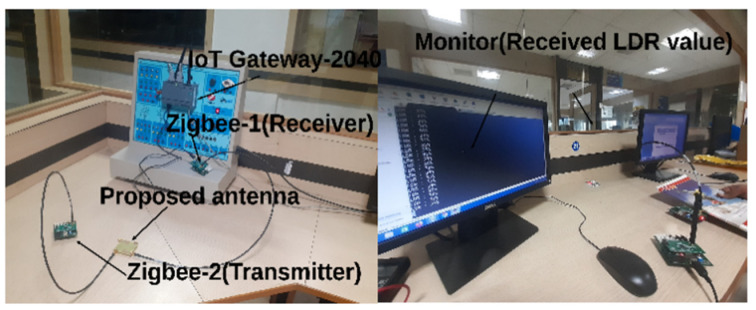
Photograph of the smart home experimental setup.

The real-time verification of the proposed DS MIMO antenna for an IoT-based smart home system is illustrated in Figure 16. Figure 17 shows the photograph of the smart home experimental setup with the proposed MIMO antenna. For real-time verification, two antennas, an IoT development kit, a monitor, and two ZigBee devices are used.

The ZigBee device has an in-built LDR sensor to detect incident light for smart home applications. The proposed antennas are connected with the first and second ZigBee devices. One antenna is acting as a transmitter, and another is working as a receiver. In this real-time IoT implementation, the LDR sensor sends the sensed data (for two different conditions, with light and without light) through the proposed antenna, which is connected to the second ZigBee device. The transmitted data is received by the receiving antenna, connected to the first ZigBee device. In order to store and retrieve data from the cloud for IoT applications, the first ZigBee device is connected to the IoT development kit, as shown in Figure 17.

Table 4 compares the proposed antenna element with the existing dual-band, triple-band, and quad-band antennas. Table 5 shows a comparison of the proposed MIMO antenna and existing MIMO antennas for IoT applications. The proposed antenna has a compact size, moderate gain, and omnidirectional radiation patterns in the operating bands, making it suitable for IoT applications.

## 5. Conclusions

The design and development of the DS MIMO antenna are presented. The proposed antenna achieves high isolation between radiating elements without the use of any additional decoupling structure. The radiators are placed orthogonally to each other on both sides of the substrate to enhance isolation. The *ECC* of the proposed DS MIMO antenna is <0.04, *DG* is >9.8 dB, TARC is <−20 dB, and CCL is ≤0.28 bit/s/Hz. The proposed antenna results show that excellent diversity, radiation performance, and almost omnidirectional radiation characteristics are achieved. In addition, the real-time realization of the quad-band DS MIMO antenna is tested for IoT-based smart home applications.

## Figures and Tables

**Table 1:** Dimensions of the quad-band antenna.

**Parameter**	*L*	*W*	*L_f_*	*W_f_*	*L*_1_	*W*_1_	*L*_2_	*L*_3_	*W*_2_	*L*_4_	*L*_5_	*L*_6_	*L*_7_
**Value** (**mm**)	30	20	7	3	8.4	1.5	8.5	4.5	1	15	12.5	3.7	8
**Parameter**	*L*_8_	*W*_3_	*L*_9_	*L*_10_	*L*_11_	*L*_12_	*W*_4_	*G*_1_	*G*_2_	*L_g_*	*L_s_*	*W_s_*	*L_t_*
**Value** (**mm**)	1.5	1.5	3.4	4.4	2.7	2.5	1.3	1.5	1	8	2	3	12

**Table 2:** Diversity performance of the proposed DS MIMO antenna.

Frequency (GHz)	Isolation (dB)	ECC_12_	ECC_13_	ECC_14_	DG_12_ (dB)	DG_13_ (dB)	DG_14_ (dB)	MEG_12_	MEG_13_	MEG_14_
1.8	>23	<0.035	<0.025	<0.023	9.83	9.88	9.85	0.997	0.985	0.987
2.4	>20	<0.015	<0.008	<0.009	9.94	9.85	9.88	0.981	0.983	0.98
3.4	>21	<0.005	<0.007	<0.003	9.98	9.94	9.99	0.999	0.997	0.995
5.4	>22	<0.003	<0.006	<0.005	10	10	10	0.994	0.984	0.982

**Table 3:** Measured TARC of the proposed DS MIMO antenna.

Frequency (GHz)	TARC_12_ (dB)	TARC_13_ (dB)	TARC_14_ (dB)
1.8	−34	−48	−45
2.4	−25	−30	−29
3.4	−38	−54	−54
5.4	−35	−36	−34

**Table 4:** Comparison of existing monopole antennas and the proposed quad-band antenna.

Ref.	Operation	Antenna Size (*L* mm × *W* mm), (*λ_L_* × *λ_W_*)	Operating Bands (GHz)
[8]	Dual-band	40 × 18, 0.32 × 0.14	2.4–2.5, 5.15–5.875
[9]	Dual-band	105 × 105, 0.30 × 0.30	0.860–0.960, 2.38–2.52
[10]	Dual-band	43 × 49, 0.33 × 0.38	2.26–2.52, 3.9–6.38
[11]	Dual-band	20 × 20, 0.57 × 0.57	8.5–9, 11–11.5
[12]	Dual-band	35 × 25, 0.13 × 0.09	1.1–2.7, 3.15–3.65
[13]	Tri-band	32 × 28, 0.24 × 0.21	2.29–2.88, 3.26–3.88, 4.17–6.07
[14]	Tri-band	45 × 65, 0.24 × 0.34	1.56–1.78, 1.96–2.16, 2.47–2.66
[15]	Tri-band	30 × 34, 0.19 × 0.21	1.9–2.1, 3.4–3.6, 5.15–5.35
[16]	Tri-band	30 × 24, 0.25 × 0.21	2.5–2.7, 3.3–3.6, 5.2–5.8
[17]	Tri-band	75 × 120, 0.23 × 0.36	0.9, 1.85, 2.4
[18]	Tri-band	36 × 29, 0.27 × 0.22	2.32–2.65, 3.21–3.34, 5.01–6.14
[19]	Tri-band	45 × 10, 0.34 × 0.07	2.3–2.69, 3.4–3.7, 5.15–5.85
[20]	Tri-band	40 × 18, 0.11 × 0.05	0.856–1.1, 1.7–2.0, 2.1–2.7
[21]	Quad-band	70 × 50, 0.32 × 0.23	1.43–1.6, 1.94–2.1, 2.42–2.57, 3.45–3.64
[22]	Quad-band	40 × 40, 0.33 × 0.33	2.47–2.54, 4.14–4.23, 5.43–5.78, 6.71–7.42
Prop.	Quad-band	30 × 20, 0.17 × 0.11	1.76–1.84, 2.37–2.56, 3.23–3.68, 5.34–5.84

**Table 5:** Comparison of existing MIMO antennas and the proposed DS MIMO antenna.

Ref.	Dimensions (*L* mm × *W* mm), (*λ_L_* × *λ_W_*)	Operating Bands (GHz)	Isolation (dB)	*ECC*	*DG* (dB)	Real Time Application Demonstration
[23]	60 × 70, 0.18 × 0.21	0.890–0.970, 1.8–1.9, 2–2.4, 2.5–2.9	15	-	-	No
[24]	65 × 70, 0.16 × 0.21	0.754–0.971, 1.65–1.83, 2–3.6, 5.14–5.6	>12	<0.19	-	Yes
[28]	75 × 150, 0.57 × 1.15	2.3–2.4, 3.3–3.6	15	<0.5	-	No
[29]	100 × 50, 1.09 × 0.54	3.3–3.6	>20	<0.009	9.8	No
[30]	110 × 55, 1.29 × 0.64	3.5, 12.5, 15	18	<0.02	9.7	No
[31]	100 × 100, 0.53 × 0.53	1.63–1.84, 2.43–2.71, 3.27–3.75	15	<0.02	10	No
[32]	56 × 56, 0.24 × 0.24	1.3–40	>22	<0.03	-	No
[33]	44 × 44, 0.20 × 0.20	1.4, 2.3, 2.45	20	-	-	No
[34]	54 × 54, 0.55 × 0.55	3.1–10.6	20	<0.009	9.7	Yes
[35]	33 × 57.5, 019 × 0.34	1.88–1.94, 2.37–2.51, 3–11	14.2	<0.047	-	No
[36]	117 × 65, 0.312 × 0.173	0.8, 1.8, 5.5–5.8, 7.2–8.9	14.5	<0.027	9.8	No
Prop.	60 × 60, 0.35 × 0.35	1.76–1.84, 2.37–2.56, 3.23–3.68, 5.34–5.84	>18	<0.03	9.8	Yes

## Data Availability

The data presented in this study are available on request from the corresponding author.
